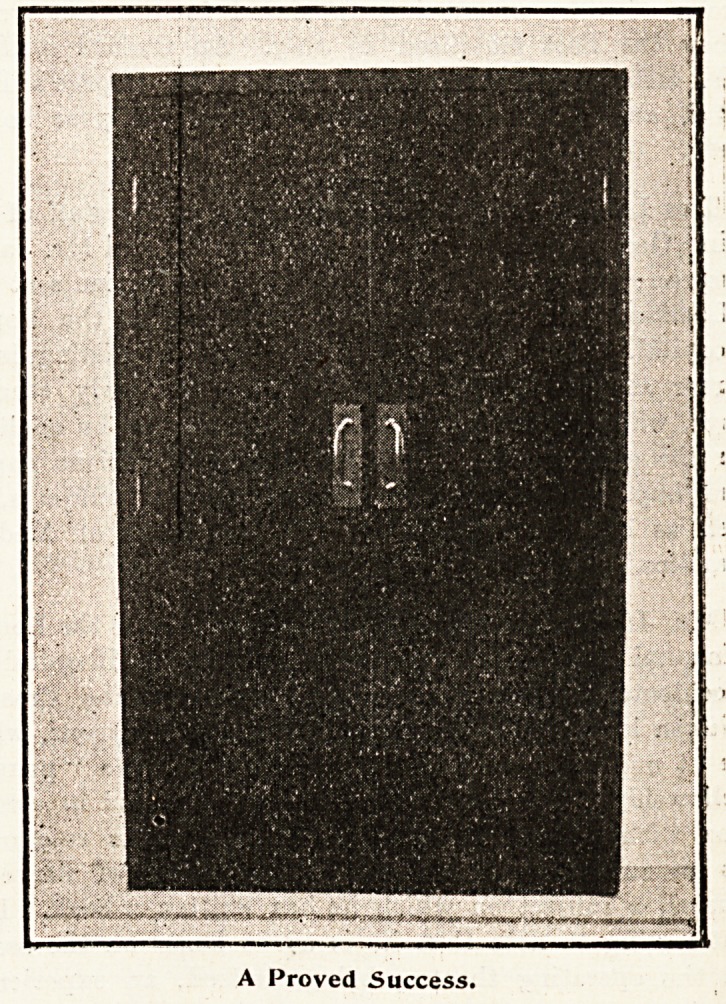# Some Home-Made Hospital Equipment: Examples from Leicester Infirmary

**Published:** 1915-01-30

**Authors:** 


### SOME HOME-MADE HOSPITAL EQUIPMENT.

### Examples from Leicester Infirmary.

We give below some further specimens of new fittings which have been made for the remodelled Children's Hospital of the Leicester Royal Infirmary. Our issue of October 10, 1914, pages 49 and 50, contained illustrations of various articles of furniture in use at this hospital, all of which, as in the case of the fittings here described, were designed and most of them made in the infirmary's works department. We should welcome a Hospital Works Department Competition. We offer a prize of one guinea for the suggestion for its organisation decided by the Editor to be the best out of a dozen or upwards if sent in for competition.

### Heating Apparatus for Theatre.

A Novel Radiator.

The above illustration shows heating apparatus specially designed for the theatre only. The large gun-metal coils can be swung from the recess, and the whole cleansed as often as necessary. The coils are controlled by a thermostat placed in the room, which keeps it at the required temperature. The approximate cost is £30.

### A Radiant Heat Novelty.

This is made of canary wood painted and enamelled, with the floor of teak. It is movable, with large Kendrick castors. The inside panels are lined with opal glass, 30 oz. The fittings are made up of two flange sockets and a piece of circular copper pipe, and are all bronzed. There are three switches arranged on the outside, one for the back lights and one for each side light. The heat and light can therefore be applied to any part of the body without unduly distressing the patient. There is also an inspection window and a place for taking temperatures. Part of the lamps are of metallic

A Canary Wood Bath—Radiant Heat.

and part of carbon filament. The seat is fixed to the floor and can be raised or lowered. The cost is £12.

### Hospital Doors.

These hospital doors are of solid teak with secret rails to prevent twisting, and ⅝-in. bolts running

A Proved Success.

right through. One such door has been fitted for eight years, and although placed in the hottest room in the institution, with a temperature sometimes approaching 90°, no sign of a crack has yet made its appearance. It will be observed that there are no mouldings whatever. The approximate cost of a door 3 ft. by 7 ft. is £4 5s.

## Figures and Tables

**Figure f1:**
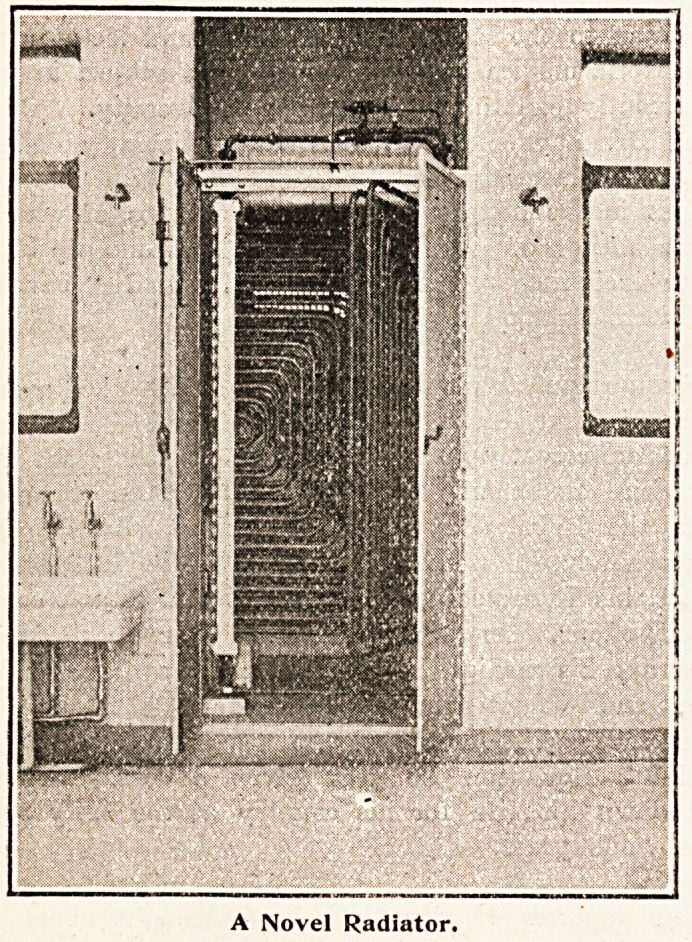


**Figure f2:**
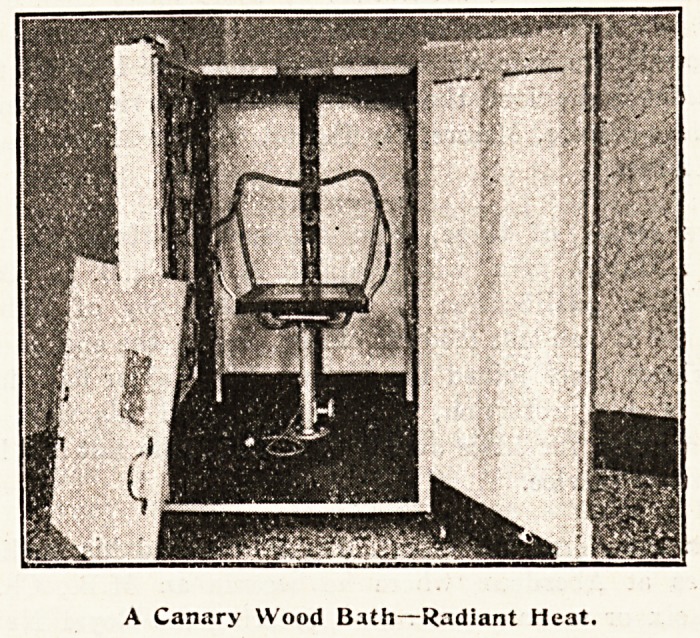


**Figure f3:**